# Evaluating the association between caffeine exposure and sleep duration and trouble sleeping in adults

**DOI:** 10.1093/sleepadvances/zpag064

**Published:** 2026-06-18

**Authors:** Patrick Viet-Quoc Nguyen, Qiaowei Lin, Shakila Meshkat, Jithin T Joseph, Reinhard Janssen, Wendy Lou, Venkat Bhat

**Affiliations:** Department of Psychiatry, Interventional Psychiatry Program, St. Michael’s Hospital, Unity Health Toronto, Toronto, Ontario, Canada; Department of Pharmacy, Centre de recherche du Centre Hospitalier de l’Université de Montréal, Montréal, Quebec, Canada; Department of Psychiatry, Interventional Psychiatry Program, St. Michael’s Hospital, Unity Health Toronto, Toronto, Ontario, Canada; Department of Psychiatry, Interventional Psychiatry Program, St. Michael’s Hospital, Unity Health Toronto, Toronto, Ontario, Canada; Department of Psychiatry, Interventional Psychiatry Program, St. Michael’s Hospital, Unity Health Toronto, Toronto, Ontario, Canada; Department of Psychiatry, Interventional Psychiatry Program, St. Michael’s Hospital, Unity Health Toronto, Toronto, Ontario, Canada; Department of Biostatistics, Dalla Lana School of Public Health, University of Toronto, Toronto, Ontario, Canada; Department of Psychiatry, Interventional Psychiatry Program, St. Michael’s Hospital, Unity Health Toronto, Toronto, Ontario, Canada; Department of Neurology, Neuroscience Research Program, St. Michael’s Hospital, Toronto, Ontario, Canada; Department of Psychiatry, University of Toronto, Toronto, Ontario, Canada; Institute of Medical Science, Temerty Faculty of Medicine, University of Toronto, Toronto, Ontario, Canada

**Keywords:** caffeine, sleep duration, epidemiology, public health, NHANES

## Abstract

**Study Objectives:**

This study examines whether caffeine is independently associated with sleep outcomes in the general population.

**Methods:**

Data from the National Health and Nutrition Examination Survey from 2005 to 2018 were analyzed, including adults aged ≥18 years who completed both dietary interviews and the Sleep Disorders Questionnaire. Caffeine exposure was estimated from a 24-h dietary recall and expressed as milligrams per day, modeled both continuously and categorically (0, 1–99, 100–399, and ≥ 400 mg/day). Sleep outcomes included self-reported sleep duration (<6 h vs. ≥6 h). Survey-weighted multivariable logistic regression models were applied to examine associations, adjusting for age, sex, race/ethnicity, education, smoking status, alcohol use, income-to-poverty ratio, and depression.

**Results:**

The sample included 37 469 adults (mean age 46.4 years; 51.7 per cent female). Compared with 0 mg/day, the adjusted odds ratios (aORs) for short sleep were 1.0 (95 per cent CI, 0.9–1.2) for 1–99 mg/day, 1.1 (95 per cent CI, 0.9–1.2) for 100–399 mg/day and 1.5 (95 per cent CI, 1.2–1.8) for ≥400 mg/day. Caffeine intake of 101–400 mg taken within 8 h of bedtime was associated with short sleep. Although significant in unadjusted analyses, the association between caffeine intake and self-reported trouble sleeping was no longer significant after multivariable adjustment (low intake: aOR 1.0 [95 per cent CI, 0.9–1.1]; moderate intake: aOR 1.0 [95 per cent CI, 0.9–1.2]; high-intake: aOR 1.0 [95 per cent CI, 0.9–1.2]).

**Conclusions:**

In this U.S. cross-sectional sample, higher caffeine intake was modestly associated with shorter self-reported sleep duration particularly when taken within 8 h of bedtime.

**Statement of Significance**

Caffeine promotes wakefulness by blocking adenosine signaling, yet its real-world impact on sleep is uncertain. Using NHANES data, we show that higher usual caffeine intake is associated with shorter sleep and greater odds of short sleep. Higher caffeine intake within 8 h before bedtime was also associated with shorter sleep duration. These population-level findings extend experimental evidence by demonstrating relevance under everyday patterns of consumption. Because caffeine timing and dose are modifiable, our results support pragmatic guidance that emphasizes mindful intake, especially limiting higher doses and avoiding late-day consumption, to improve sleep health. This work highlights actionable targets for public health messaging and motivates prospective studies testing whether reducing caffeine exposure lengthens sleep and mitigates downstream risks.

## Graphical Abstract

**Graphical Abstract:**
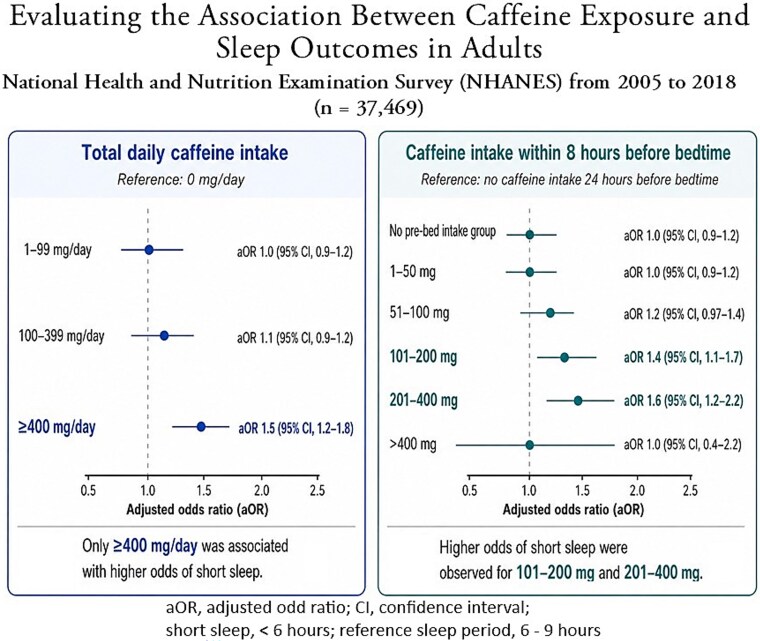

## Introduction

Coffee is a very popular and broadly consumed beverage worldwide, enjoyed not only for its taste and aroma, but also for its effects as a stimulant on the central nervous system [1]. Its primary psychoactive constituent is caffeine, which is now added to many beverages and foods for its stimulant properties.

Caffeine directly interacts with the biological systems regulating sleep and wakefulness. Under normal conditions, adenosine accumulation during prolonged wakefulness increases sleep pressure through A_1_ and A_2_A receptors [2, 3]. By antagonizing these receptors, caffeine promotes wakefulness, reduces perceived sleepiness, and delays the onset of sleep. However, this same mechanism can contribute to sleep problems, including difficulty initiating sleep, shorter sleep duration, and lighter or fragmented sleep, particularly when consumed later in the day [4].

The consequences of this pharmacological action on sleep have been well characterized at the individual level. In a meta-analysis, caffeine consumption was associated with a 45-min reduction in total sleep time, a 9-min increase in sleep onset latency, and 12 min more wake time after sleep onset; sleep efficiency was reduced by 7 per cent [5]. These effects are dose dependent: in a randomized placebo-controlled trial, high-dose caffeine (400 mg), but not low-dose caffeine (100 mg), significantly delayed sleep onset and increased sleep fragmentation, even when ingested up to 12 h before bedtime [6]. Timing is also a critical determinant of its impact, as caffeine’s half-life of 3–7 h means that evening consumption is substantially more disruptive to sleep than intake earlier in the day [7].

Randomized trials have established acute effects of experimentally administered caffeine on sleep [5], but it is less clear how usual daily caffeine intake relates to self-reported sleep at the population level across age groups and sociodemographic strata. Clarifying this relationship is critical for shaping public health education initiatives on sleep health and the consumption of coffee and other caffeine-containing foods and beverages. By leveraging 14 years of the National Health and Nutrition Examination Survey (NHANES) data, we aim to examine cross-sectional associations between usual daily caffeine intake and self-reported sleep duration and sleep complaints in the general U.S. adult population.

## Materials and methods

### Study population

The NHANES, conducted by the National Center for Health Statistics (NCHS) at the Centers for Disease Control and Prevention (CDC), is an ongoing series of biennial cross-sectional surveys designed to assess the health and nutritional status of community-dwelling U.S. residents. Using a complex, multistage, stratified probability sampling design, NHANES selects participants to be representative of the noninstitutionalized U.S. population. Each year, ~5000 individuals complete household interviews followed by standardized physical examinations and laboratory assessments in mobile examination centers. Data are collected continuously and released in 2-year cycles beginning in 1999, with detailed methodology available on the NHANES website [8]. The NCHS Research Ethics Review Board approved all NHANES protocols and written informed consent was obtained from all participants. The present study is a secondary analysis of de-identified, publicly available data which did not necessitate additional institutional ethics approval.

We used data from survey cycles 2005–2018 for this analysis. The analytic sample was restricted to adults aged ≥18 years who completed the caffeine intake items from the dietary interview (DR1TOT) and the Sleep Disorders Questionnaire (SLQ).

### Exposures

Dietary intake was assessed using two interviewer-administered 24-h recalls. Total caffeine intake (mg/day) was estimated using the NHANES Day 1 Total Nutrient Intakes variable DR1ICAFF, which reflects caffeine intake (mg) from all foods and beverages reported during the 24-h dietary recall. Mean intake was analyzed both continuously and categorically using cutoffs of 0, 1–99 mg/day, 100–399 mg/day, and ≥ 400 mg/day, selected to align with doses commonly used in prior experimental studies on caffeine and sleep [6]. After ingestion, caffeine has a half-life of ~1.5–9.5 h, and its pharmacological effects generally decline as plasma concentrations decrease. Because meaningful exposure may persist for several hours, timing of intake is relevant to sleep. To assess this, we linked the reported time of each eating occasion from the dietary interview with item-level caffeine intake and calculated total caffeine consumed within 8 and 12 h before usual bedtime. Usual bedtime was obtained from SLQ300 was only available for NHANES Cycle 2015–2018; for other cycles, it was imputed using the mean bedtime for participants of the same age and sex category. Caffeine exposure was categorized as No daily caffeine (0 mg/day), No pre-bed caffeine (0 mg within 8 or 12 h before bedtime but earlier intake), or pre-bed intake of 1–50, 51–100, 101–200, 201–400, or > 400 mg.

### Outcomes

Sleep characteristics were obtained from the SLQ. Sleep duration was assessed by self-report using the question, “How much sleep do you usually get at night on weekdays or workdays?” Participants reported their usual number of hours of sleep per night. We defined short sleep as <6 h versus 6–9 h per night. Participants with >9 h of sleep were excluded from the reference group because long sleep is associated with underlying health conditions and may bias comparisons [9]. Trouble sleeping was defined as a “yes” response to the question, “Have you ever told a doctor or other health professional that you had trouble sleeping?”. The sleep questionnaire was administered during the in-home interview, whereas caffeine intake was assessed during the dietary interview conducted in the mobile examination center ~2 weeks later.

### Covariates

Covariates were selected a priori based on prior evidence for associations with caffeine use and sleep. Demographic variables included age, sex, race/ethnicity, education, and family poverty-to-income ratio (PIR). Age was modeled continuously and categorized as 18–39, 40–64, and ≥ 65 years for interaction and stratified analyses. Sex, race/ethnicity, and education were grouped using standard NHANES categories. PIR was categorized as low (<1), middle (1–4), or high (>4) [10]. Lifestyle factors included smoking status, defined as current use (yes/no) and alcohol consumption was classified as never (no lifetime alcohol use), light (1–11 drinking occasions in the past year), moderate (drinking on ≥1 occasion per month but no more than twice per week), and heavy (drinking on more than two occasions per week). Depressive symptoms were assessed with the 9-item Patient Health Questionnaire and included as a binary covariate (PHQ-9 ≥ 10 vs. <10) [11]. Self-reported physician-diagnosed diabetes and heart failure were coded as binary (yes vs. no). Daytime sleepiness was assessed using SLQ120: “In the past month, how often did you feel excessively or overly sleepy during the day?”

For descriptive purposes (not included in models), we also reported body mass index (BMI, kg/m^2^; <18.5 underweight, 18.5–24.9 normal weight, 25.0–29.9 overweight, and ≥ 30.0 obesity) [12] and self-reported physician diagnoses of hypertension. Use of hypnotic medications (benzodiazepines and Z-drugs: zolpidem, zaleplon, and eszopiclone) was identified and coded as yes if any of these medications were reported and no otherwise in the prescription medication file.

### Statistical analyses

All analyses accounted for the complex NHANES design using the survey package in R [13] (version 4.4.0), applying sampling weights, strata, and primary sampling units as recommended by the NCHS. The dietary day one sample weight (WTDRD1) was used for analyses and divided by seven to combine seven cycles. Descriptive statistics were generated for the overall sample and by caffeine intake category. Survey-weighted means and standard deviations were reported for continuous variables, and survey-weighted proportions for categorical variables.

Survey-weighted multivariable logistic regression models were used to estimate associations between categorical caffeine intake and presence of trouble sleeping, sleep duration (<6 vs. 6–9 h). Three models were fitted for all analyses: an unadjusted model including only the exposure; a partially adjusted model including the exposure and the nine covariates described above, excluding heart failure; and a fully adjusted model including the exposure and all ten covariates described above. Heart failure was excluded from the partially adjusted model because this variable was assessed only among participants aged 20 years and older, whereas the study included participants aged 18 years and older. Effect modification by age group (18–39, 40–64, and ≥ 65 years) was examined by including interaction terms between caffeine intake and age group in the logistic models. As none of the interaction *p*-values in the adjusted models was <0.05, subgroup analyses were not conducted.

To account for potential confounding due to the use of caffeine as a compensatory strategy for daytime sleepiness, we conducted sensitivity analyses restricted to NHANES cycles in which SLQ120 was available: 2005–2006, 2007–2008, 2015–2016, and 2017–2018. In these analyses, the dietary Day 1 sample weight was divided by four to account for the four included cycles. We refitted the models for total daily caffeine intake and caffeine intake within 8 and 12 h before bedtime. Model 1 was unadjusted, Model 2 included the fully adjusted covariates from the primary analysis, and Model 3 additionally adjusted for daytime sleepiness.

To assess potential non-linear associations between continuous total daily caffeine intake and trouble sleeping, short sleep (<6 h), we fitted survey-weighted logistic regression models among participants reporting non-zero total daily caffeine intake. Caffeine intake was modeled using natural cubic splines with 1–4 degrees of freedom (df) and compared model fit using Akaike’s information criterion (AIC). As models with ≥2 df did not materially improve fit over the 1-df model, we retained a spline with 1 df, mathematically equivalent to modeling caffeine intake as a linear term on the log-odds scale in the final analyses. Using the fully adjusted model, we then estimated the predicted probability of short sleep across the 0th–95th percentile of caffeine intake (1–510 mg/day for the trouble sleeping sample and 1–513 mg/day for the sleep duration sample; holding covariates at their mean or reference values). For graphical display, we identified the caffeine intake values corresponding to selected absolute risk levels within the observed range of predicted probabilities for each outcome.

Model fit was assessed using standard regression diagnostics; spline analyses were exploratory. Adjusted models were based on complete cases for exposures, outcomes, and covariates. Multiple imputation was not undertaken because of the complexity of the survey design and scope of the project; complete-case analysis may bias estimates if missingness is related to both caffeine intake and sleep outcomes. All tests were two-sided, with *p* < .05 considered statistically significant.

## Results

### Demographic and descriptive results

**Figure 1:**
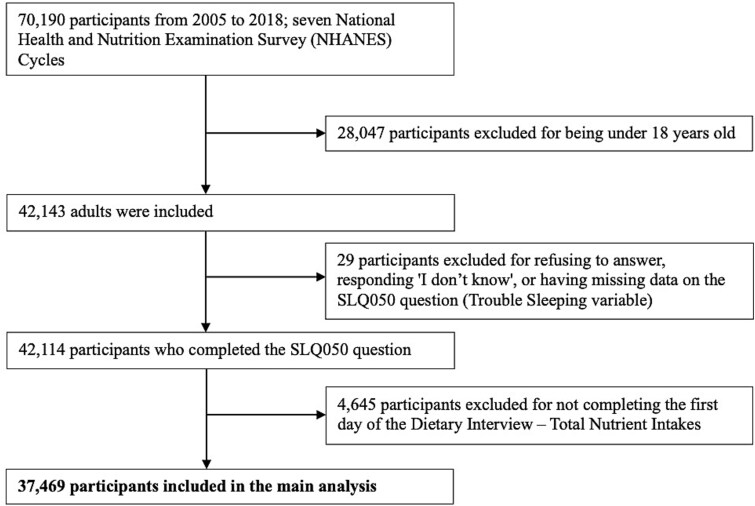
Study flowchart showing participant selection and exclusions.

Participant selection and exclusions are summarized in Figure 1, which traces the sample from initial NHANES respondents to the final analytic cohort. The final sample included 37 469 adults (mean age 46.4 ± 17.6 years), of whom 51.7 per cent (n = 19 234) were female. Overall, 12.4 per cent of participants reported no caffeine, 33.0 per cent had low caffeine intake (1–99 mg), 44.3 per cent had moderate intake (100–399 mg), and 10.3 per cent had high-intake (≥ 400 mg). Sociodemographic, lifestyle, and clinical characteristics are presented in Table 1. Hypnotics use increased across caffeine categories, from 3.8 per cent (n = 171) in the zero-caffeine group, 4.3 per cent (n = 535) in the low-intake group to 5.2 per cent (n = 694) in the moderate-intake group and 6.0 per cent (n = 174) in the high-intake group. Hypnotic-use data were missing for 0.6 per cent of participants (n = 258). The prevalence of hypnotic use differed significantly across caffeine groups (*p* < .001).

**Table 1:** Sociodemographic and health characteristics of the population

**Characteristics**	**Daily caffeine intake**			
	0 mg (n = 5776)	1–99 mg (n = 14, 090)	100–399 mg (n = 14 720)	≥400 mg (n = 2883)
**Demographics**				
Age in years, mean (SD)	40.0 (17.8)	44.9(18.8)	48.6 (16.8)	49.8 (13.8)
Female sex, % (n)	50.90 (3002)	57.5 (7924)	50.3 (7177)	40.3 (1131)
Race/ethnicity, % (n)				
Mexican American	11.8 (963)	11.7(2704)	7.1 (2171)	3.2 (230)
Other Hispanic	6.4 (485)	6.8 (1490)	4.9 (1404)	2.4 (157)
Non-Hispanic White	45.3 (1359)	57.9 (4701)	74.3(7473)	87.2 (2174)
Non-Hispanic Black	26.9 (2293)	14.8 (3543)	6.9 (2223)	2.5 (185)
Other races	9.7 (676)	8.8 (1652)	6.9 (1449)	4.9 (164)
Education, % (n)				
Less than 9th grade	6.2 (579)	6.8 (1676	4.1 (1282)	3.5 (186)
9–11th grade	14.8 (1037)	12.3 (2293)	9.6 (2018)	12.1 (428)
High school graduate	24.9 (1425)	24.3 (3335)	24.4 (3.464)	24.0 (725)
Some college or associate degree	31.6 (1558)	30.1 (3636)	30.4 (4258)	34.6 (946)
College graduate or higher	22.5 (992)	26.5 (2.858)	31.6 (3574)	25.9 (590)
Missing data	2.6 (185)	1.5 (292)	0.6 (124)	0.1 (8)
Income-to-Poverty Ratio, % (n)				
Low (<1.0)	21.0 (1489)	16.2 (3.092)	11.1 (2426)	11.8 (546)
Middle (1.0–4.0)	47.9 (2773)	47.0 (6.838)	44.6 (7221)	43.8 (1386)
High (>4.0)	23.2 (968)	28.9 (2803)	38.0 (3954)	38.8 (778)
Missing data	7.8 (546)	8.0 (1357)	6.3 (1119)	5.5 (173)
**Lifestyle & Anthropometrics**				
Body mass index, kg/m^2^				
<18.5	1.9 (115)	2.0 (282)	1.4 (215)	1.4 (54)
18.5–24.9	30.2 (1701)	30.6 (4060)	27.5 (3879)	25.3 (740)
25.0–29.9	27.1 (1633)	31.0 (4478)	34.0 (4967)	34.5 (951)
≥30.0	38.7 (2226)	35.4 (5084)	36.4 (5531)	38.3 (1115)
Missing data	2.2 (101)	1.1 (186)	0.6 (128)	0.5 (23)
Current smoker, % (n)	15.8 (908)	14.4 (1987)	20.5 (3149)	40.2 (1260)
Missing data	4.5 (398)	2.9 (668)	1.3 (307)	0.5 (20)
Current alcohol use, % (n)				
Never	31.8 (1968)	28.3 (4767)	21.2 (3908)	22.2 (775)
Light (>0–3 drinks/week)	15.8 (965)	18.0 (2570)	18.0 (2776)	18.0 (553)
Moderate (≤7 drinks/week for women; ≤14 for men)	32.3 (1590)	34.9 (4081)	36.4 (4.804)	33.5 (905)
Heavy (>7/week for women; >14 for men)	11.9 (616)	11.0 (1249)	19.2 (2216)	21.7 (515)
Missing data	8.3 (637)	7.9 (1423)	5.3 (1016)	4.7 (135)
**Clinical**				
Diabetes, % (n)	9.3 (655)	8.8 (1685)	9.3 (1876)	10.3 (360)
Missing data, n	4	5	12	1
Hypertension, % (n)	21.1 (1392)	24.9 (3838)	26.0 (4259)	26.0 (839)
Missing data	0.2 (13)	0.3 (49)	0.2 (45)	0.2 (5)
Congestive Heart failure, % (n)	2.4 (168)	2.4 (455)	2.1 (425)	2.9 (110)
Missing data	7.7 (657)	5.2 (1096)	2.1 (479)	0.7 (32)
Depressive symptoms (PHQ-9 ≥10), % (n)	8.1 (451)	7.4 (1111)	6.9 (1129)	10.1 (334)
Missing data	6.7 (409)	6.7 (1084)	4.9 (884)	4.5 (129)
Mean caffeine intake, mean (SD)	0.0 (0)	41.2 (31.2)	210 (80.5)	642.1 (333.2)

Note: Categorical characteristics reported as unweighted frequency and weighted percent.

Continuous characteristics reported as weighted mean and standard deviation.

### Association between caffeine intake and self-reported trouble sleeping

**Figure 2:**
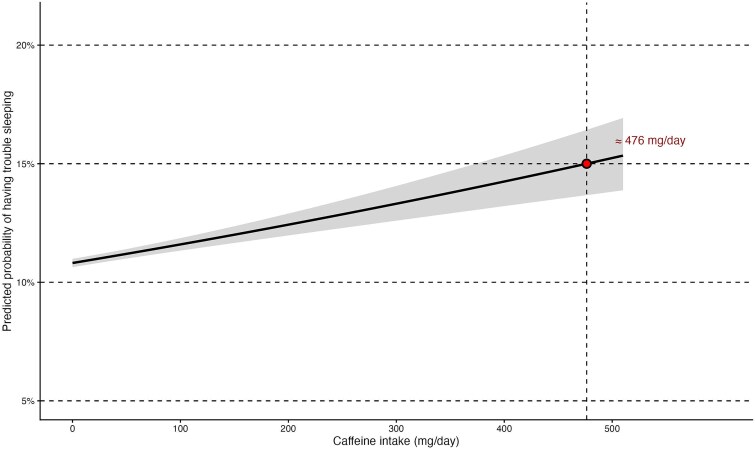
Modeled dose–response relationship between caffeine intake and trouble sleeping.

Overall, 27.2 per cent (n = 9297) reported having told a clinician they had trouble sleeping. The prevalence increased across caffeine intake categories: 23.7 per cent (n = 1237) in the zero-caffeine group, 25.2 per cent (n = 3272) in the low-intake group, 28.6 per cent (n = 3864) in the moderate-intake group, and 32.1 per cent (n = 924) in the high-intake group. Although weighted unadjusted comparisons were significant for the moderate- and high-intake groups (*p* < .001; Table 2), the associations were no longer significant after multivariable adjustment: aOR 1.0 (95 per cent CI, 0.9–1,1) for low intake, aOR 1.0 (95 per cent CI, 0.9–1.2) for moderate intake and aOR 1.0 (95 per cent CI, 0.9–1.2) for high-intake. The interaction between age and caffeine intake was not statistically significant (Table S1). The modeled dose–response relationship between caffeine intake and the probability of having trouble sleeping is shown in Figure 2. In the fully adjusted model, the predicted probability of having trouble sleeping increased slightly with higher caffeine intake, rising from ~11 per cent at very low intake to ~15 per cent at ~476 mg/day.

**Table 2:** Associations between total dietary caffeine intake and sleep outcomes

Total dietary caffeine intake	Trouble sleeping (Yes vs. No) (OR [95% CI])			Sleep duration (< 6 h vs. 6–9 h) (OR [95% CI])		
	Model 1	Model 2	Model 3	Model 1	Model 2	Model 3
Low (1–99 mg/day)	1.08 (0.97, 1.21)	0.99 (0.87,1.13)	0.98 (0.86,1.13)	0.86^a^ (0.75,0.99)	1.03 (0.89,1.19)	1.04 (0.89,1.20)
Moderate (100–399 mg/day)	1.29^a^ (1.16,1.43)	1.02 (0.91,1.15)	1.02 (0.90,1.15)	0.79^a^ (0.70,0.90)	1.05 (0.91,1.21)	1.05 (0.91,1.21)
High (≥400 mg/day)	1.53^a^ (1.34,1.74)	1.02 (0.87,1.20)	1.02 (0.86,1.20)	1.17 (0.98,1.41)	1.46^a^ (1.18,1.81)	1.47^a^ (1.18,1.82)

Note: OR = odds ratio; CI = confidence interval. Dietary caffeine intake is the exposure variable, the reference level is “No Caffeine (0 mg/day)”. Sleep outcomes were modeled separately (N = 37 469 for trouble sleeping, N = 35 291 for sleep duration). The reference level for sleep duration is (6–9 h), the reference level for trouble sleeping is “No.” Model 1 was unadjusted and included dietary caffeine intake as the independent variable only. Model 2 was adjusted for age (continuous), sex, race, education, PIR, smoking status, alcohol consumption, depressive symptoms, and diabetes. Model 3 was additionally adjusted for heart failure.

a Indicate statistical significance (95% CI excluding 1).

In timing analyses, caffeine intake within 12 h before bedtime was associated with higher unadjusted odds of trouble sleeping at all intake levels, but no association remained significant after adjustment. Similarly, within 8 h before bedtime, several categories showed elevated unadjusted odds, particularly the no pre-bed, 1–50 mg, 51–100 mg, 101–200 mg, and 201–400 mg groups; however, all associations were attenuated and no longer statistically significant in multivariable models (Tables S2 and S3). No significant interaction was observed between age group and caffeine intake for trouble sleeping (Tables S4 and S5).

In sensitivity analyses restricted to four cycles, 20 986 participants were included in trouble-sleeping analysis, and the pattern of findings was similar. Total daily caffeine intake and caffeine intake within 8 or 12 h before bedtime were associated with trouble sleeping in several unadjusted models; however, these associations were attenuated after multivariable adjustment and remained non-significant after additional adjustment for daytime sleepiness (Tables S6–S8).

### Association between caffeine intake and sleep duration

The survey-weighted mean sleep duration was 7.2 h overall and in the zero-caffeine, low-intake, and moderate-intake groups, compared with 6.9 h in the high-intake group. Short sleep (<6 h) occurred in 12.4 per cent (n = 839), 11.0 per cent (n = 1861), 10.4 per cent (n = 1879), and 14.8 per cent (n = 498) of participants in the zero-caffeine, low-, moderate-, and high-intake groups, respectively. After excluding 2072 participants with sleep duration outside the 6–9 h reference range and 106 with missing sleep-duration data, 35 291 participants were included in the short-sleep analysis.

**Figure 3:**
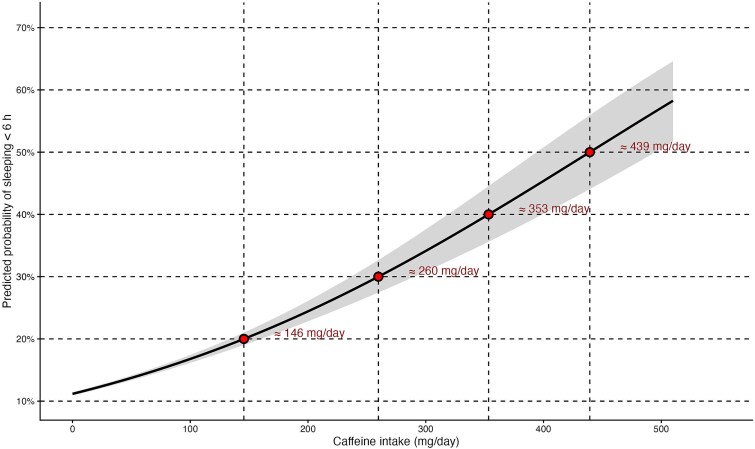
Modeled dose–response relationship between daily caffeine intake and short sleep (<6 h).

In the adjusted model, only high caffeine intake was associated with greater odds of short sleep (adjusted odds ratio [aOR] 1.5; 95 per cent CI, 1.2–1.8; Table 2). The modeled dose–response relationship between caffeine intake and the probability of sleeping <6 h is shown in Figure 3. In the fully adjusted model, the predicted probability of short sleep increased from ~11 per cent at very low intake to ~50 per cent at ~439 mg/day, and to nearly 58 per cent at the upper end of intake (~513 mg/day).

In timing analyses, caffeine consumed within 12 h of bedtime was not associated with sleep duration (Table S2). In contrast, when exposure was restricted to the 8-h pre-bedtime window, higher intake categories were associated with greater odds of short sleep. Compared with no intake, aORs were 1.0 (95 per cent CI, 0.9–1.2) for the no pre-bed intake and 1–50 mg, 1.2 (95 per cent CI, 0.97–1.4) for 51–100 mg, 1.4 (95 per cent CI, 1.1–1.7) for 101–200 mg, 1.6 (95 per cent CI, 1.2–2.2) for 201–400 mg, and 1.0 (95 per cent CI, 0.4–2.2) for >400 mg (Table S3). No significant interaction was observed between age group and caffeine intake for short sleep (Tables S4 and S5).

In the corresponding sensitivity analyses for short sleep, 19 317 participants were included. High total daily caffeine intake remained associated with greater odds of short sleep after additional adjustment for daytime sleepiness (aOR = 1.46; 95 per cent CI, 1.04–2.06; Table S6). Caffeine intake within 12 h before bedtime was not significantly associated with short sleep after multivariable adjustment (Table S7). For caffeine intake within 8 h before bedtime, the 101–200 mg/day category remained associated with short sleep after adjustment for daytime sleepiness (aOR = 1.58; 95 per cent CI, 1.15–2.17), whereas the 201–400 mg/day category was not statistically significant in this restricted 4-cycle sample before or after adjustment for daytime sleepiness (Table S8).

## Discussion

In this sample of U.S. adults, higher caffeine intake was associated with an increased likelihood of short sleep, whereas moderate intake showed no meaningful relationship. This pattern is broadly consistent with prior work, including findings from Halldorsson *et al.*, who reported that adolescents with higher caffeine consumption were more likely to obtain <6 h of sleep [14].

Our findings indicate that caffeine intake within 12 h before bedtime was not associated with shorter sleep duration, whereas intake of 101–400 mg within 8 h was associated with shorter sleep. No association was observed for intake >400 mg within 8 h, although this estimate was imprecise due to the small sample size and should be interpreted cautiously. Most results remained significant after adjustment for daytime sleepiness, suggesting that the observed associations were not fully explained by caffeine use as a compensatory response to sleepiness. The only association that lost statistical significance had a similar effect estimate, but a wider confidence interval due to the smaller sample size, suggesting reduced precision rather than a clear change in the direction or magnitude of the association. These results are consistent with experimental evidence. Gardiner *et al*. showed that a 400-mg dose had no effect on total sleep time when taken 12 h before bedtime, a modest effect at 8 h, and a significant reduction when taken 4 h before bedtime, while a 100-mg dose had minimal impact at any time point [6]. Our population-based findings extend these results by suggesting that intermediate doses (100–400 mg) may also be associated with shorter sleep when consumed close to bedtime. However, given the observational design, these associations should be interpreted cautiously and confirmed in controlled trials examining a broader range of doses. Taken together, experimental and epidemiological evidence suggests that lower caffeine intake may have limited effects on sleep duration, whereas higher intake, particularly when consumed closer to bedtime, is more likely to be associated with short sleep. Nonetheless, causal inferences cannot be made due to the cross-sectional nature of our data.

Interestingly, although participants with higher caffeine intake were more likely to report having told a clinician that they had trouble sleeping in unadjusted analyses, this association was no longer significant after adjustment for sociodemographic and clinical factors. This pattern suggests that the unadjusted relationship was largely explained by underlying differences correlated with both caffeine use and sleep complaints, rather than by caffeine intake itself. It is also important to note that self-reported short sleep and reporting sleep difficulties to a clinician reflect distinct constructs. Qualitative studies indicate that individuals with insomnia symptoms often delay seeking medical care, with estimated delays of several years between symptom onset and clinical consultation [15]. Furthermore, a subset of short sleepers may represent “natural short sleepers,” who maintain short sleep without daytime impairment [16]. Such individuals would not necessarily seek medical care and would not be expected to vary systematically by caffeine intake; if anything, they may be more common in lower-intake groups, as caffeine is frequently used to counteract insomnia-related daytime symptoms [17]. Although NHANES cannot distinguish these phenotypes, these considerations help explain why only a portion of adults with <6 h of sleep report “trouble sleeping” to a clinician.

Current consensus recommends at least 7 h of sleep for adults across age groups [18]. We selected a cutoff of <6 h rather than <7 h because shorter sleep duration at this level has been more strongly associated with adverse cardiovascular outcomes and impaired functional capacity [19]. We selected covariates a priori based on literature on determinants of sleep and caffeine use. Important potential confounders were unavailable, including chronotype, shift work, and detailed occupational schedules, which may influence both caffeine consumption and sleep. We did not adjust for hypnotic use because short sleep may prompt hypnotic initiation independently of caffeine; because adjusting for a variable on the causal pathway could introduce overadjustment and bias results toward the null. Likewise, the PHQ-9 includes a sleep item; adjusting for the total PHQ-9 score risks partial control of the outcome itself. Notably, the association between caffeine intake and short sleep remained significant even after adjusting for probable depression (PHQ-9 ≥ 10), supporting the robustness of our findings. This is consistent with evidence showing that sleep disturbances can persist independently of depressive symptoms, including the well-documented phenomenon of residual insomnia in individuals whose mood symptoms have otherwise remitted, indicating that sleep problems are not solely attributable to depression [20].

We observed a higher prevalence of hypnotic use among participants with high caffeine intake. Although our cross-sectional data cannot determine whether higher caffeine intake precedes, follows, or is unrelated to hypnotic initiation, this co-occurrence raises the hypothesis of a potential “prescribing cascade,” [21] whereby stimulant-related sleep disturbance could contribute to subsequent hypnotic prescribing. Our analyses were not designed to test this hypothesis, and longitudinal studies with time-stamped medication data would be required to evaluate it. Hypnotic use is not benign; these medications carry risks of dependence and daytime sedation in the general population, and, in older adults, they are associated with cognitive impairment and a higher risk of falls [22–25]. Considering these potential harms, prospective observational studies, ideally with time-stamped exposure data, are warranted to determine whether higher caffeine intake predicts subsequent hypnotic initiation and related adverse outcomes.

Sociodemographic differences were observed in age, sex, race/ethnicity, education, income, smoking status, and daily caffeine intake; analyses were adjusted accordingly to account for these factors.

This study has notable strengths. We leveraged NHANES, a CDC program with rigorous, standardized protocols and a complex sampling design, allowing population-level inference for community-dwelling U.S. adults. Pooling multiple cycles yielded a large sample and rich phenotyping, enabling subgroup analyses and adjustment for a broad array of sociodemographic, lifestyle, clinical, and mental-health covariates.

Several limitations merit caution. NHANES is nationally representative surveys, but the exclusion of 11 per cent of adults because of missing data could have introduced selection bias reducing the representativeness of the sample. Because exposure and outcomes were assessed at the same time, we cannot establish temporality or causality, and our findings should be interpreted as associations rather than evidence that caffeine intake reduces sleep. Sleep duration, sleep problems, and dietary intake were self-reported and are therefore subject to recall and reporting biases. For participants with missing bedtime data, imputation was required, which may have led to misclassification of caffeine exposure within the 8- and 12-h pre-bedtime windows. This could have reduced precision and biased estimates. It would also have been relevant to adjust the analyses for other sleep-related variables, such as daytime sleepiness. However, these variables could not be included because they were not available in the 2009–2014 survey cycles. Although we did not directly assess insomnia or other psychiatric or neurologic disorders, our findings suggest that sleep duration may be adversely affected at higher levels of caffeine intake. Sleep duration was estimated from the self-reported interval between usual bedtime and wake time. This measure reflects “time in bed” and may overestimate true sleep duration because it does not capture sleep latency, nocturnal awakenings, or time awake in bed. This overestimation would likely cause nondifferential misclassification, biasing associations toward the null. Reverse causation remains a key concern. Short sleepers may increase caffeine to counter daytime sleepiness, which could exaggerate the observed association in a cross-sectional design where exposure and outcome are measured simultaneously, and directionality cannot be established. However, reverse causation cannot be fully excluded because we lacked information on the timing of caffeine intake, sources/formulations (e.g. energy drinks vs coffee), and contextual factors (e.g. shift work, chronotype), all of which could differentially link short sleep to subsequent caffeine use. Finally, despite extensive adjustment, residual confounding (e.g. chronotype, work schedules, timing of caffeine, co-ingestion of other stimulants) may remain, and our categorical cut points for short sleep and caffeine intake could mask important heterogeneity.

In summary, this study extends experimental evidence into a large sample and supports a dose-dependent association between caffeine intake and shorter sleep duration. Given that sleep, alongside nutrition and physical activity, is a fundamental pillar of health [26], these findings have meaningful public health relevance. Prospective cohort studies and pragmatic trials of caffeine reduction are needed to determine causality, quantify effect sizes, and inform evidence-based guidance on caffeine consumption and sleep health.

## Supplementary Material

Supplementary_Materials_zpag064

## Data Availability

All data analyzed in this study are publicly available through the National Health and Nutrition Examination Survey (NHANES), a program of the National Center for Health Statistics, Centers for Disease Control and Prevention. The datasets supporting the findings of this study can be accessed at NHANES website. No new data were generated for this study. The statistical code used for the analyses is available from the corresponding author upon reasonable request.
