# Supplementary material for: Evaluating the association between caffeine exposure and sleep duration and trouble sleeping in adults
Source: Sleep Adv. 2026 Jun 18;7(3):zpag064. doi: 10.1093/sleepadvances/zpag064 (PMC13363248; doi:10.1093/sleepadvances/zpag064)
Supplement: Supplementary_Materials_zpag064 [file supplementary_materials_zpag064.docx]

**Article title:**  **Evaluating the Association Between Caffeine Exposure and
Sleep Duration and Trouble Sleeping in Adults**

**Author list:** Patrick Viet-Quoc **Nguyen**^1,2*^, Qiaowei **Lin**^1*^, Shakila **Meshkat**^1^, Jithin T **Joseph**^1^, Reinhard **Janssen**^1^, Wendy **Lou**^3^, Venkat **Bhat**^1,4,5,6**^.

**Affiliations:**

^1^Interventional Psychiatry Program, St. Michael’s Hospital, Unity Health Toronto, Toronto, Ontario, Canada

^2^Centre de recherche du Centre Hospitalier de l’Université de Montréal, Montréal, Quebec, Canada

^3^Department of Biostatistics, Dalla Lana School of Public Health, University of Toronto, Toronto, Ontario, Canada

^4^Neuroscience Research Program, St. Michael’s Hospital, Toronto, Ontario, Canada

^5^Department of Psychiatry, University of Toronto, Toronto, Ontario, Canada

^6^Institute of Medical Science, Temerty Faculty of Medicine, University of Toronto, Toronto, Ontario, Canada

***co-first authors**

****Corresponding author:**

Venkat Bhat, MD MSc

Interventional Psychiatry Program, St. Michael’s Hospital, 193 Yonge Street 6-013, Toronto, Ontario, Canada, M5B 1M8

Phone: (416) 360-4000 x76404, Fax: (416) 864-5996, [**venkat.bhat@utoronto.ca**](mailto:venkat.bhat@utoronto.ca)

ORCID: 0000-0002-8768-1173

**Table S1.** Age interaction models for associations between Total Dietary Caffeine Intake and Sleep outcomes

| Exposures | Trouble sleeping  (Yes vs. No)  (OR [95% CI]) | | | Sleep duration  (< 6 hours vs. 6-9 hours)  (OR [95% CI]) | | |
| --- | --- | --- | --- | --- | --- | --- |
|  | Model 1 | Model 2 | Model 3 | Model 1 | Model 2 | Model 3 |
| Low intake  x Aged 40-64 | 0.89  (0.69,1.16) | 0.95  (0.71,1.27) | 0.99  (0.73,1.33) | 0.98  (0.71,1.35) | 1.14  (0.78,1.66) | 1.12  (0.77,1.63) |
| Moderate intake  x Aged 40-64 | 0.96  (0.75,1.22) | 1.12  (0.86,1.46) | 1.14  (0.87,1.50) | 0.85  (0.64,1.13) | 1.19  (0.86,1.64) | 1.20  (0.87,1.65) |
| High intake  x Aged 40-64 | 0.71^*^  (0.51,0.98) | 0.91  (0.64,1.28) | 0.92  (0.65,1.30) | 0.88  (0.59,1.29) | 1.14  (0.74,1.76) | 1.14  (0.74,1.75) |
| Low-intake  x Aged ≥ 65 | 1.16  (0.86,1.56) | 1.16  (0.82,1.64) | 1.21  (0.85,1.71) | 1.20  (0.88,1.65) | 1.18  (0.80,1.73) | 1.17  (0.79,1.72) |
| Moderate intake  x Aged ≥ 65 | 0.90  (0.67,1.22) | 1.04  (0.75,1.43) | 1.08  (0.78,1.49) | 0.95  (0.67,1.34) | 1.15  (0.80,1.66) | 1.18  (0.81,1.72) |
| High intake  x Aged ≥ 65 | 0.69  (0.45,1.06) | 0.88  (0.54,1.45) | 0.88  (0.54,1.46) | 0.98  (0.58,1.66) | 1.11  (0.63,1.95) | 1.12  (0.64,1.96) |

Note: OR = odds ratio; CI = confidence interval. The reference level of dietary caffeine intake is ‘No Caffeine (0 mg/day)’. The reference level of the age group is ‘aged 18-39’. Sleep outcomes were modeled separately. The reference level for sleep duration is (6-9 hours), the reference level for trouble sleeping is ‘No’. Model 1 was unadjusted and included dietary caffeine intake as the independent variable only. Model 2 was adjusted for sex, race, education, PIR, smoking status, alcohol consumption, depressive symptoms, and diabetes. Model 3 was additionally adjusted for heart failure.

* indicate statistical significance (95% CI excluding 1).

**Table S2.** Associations between Caffeine Intake within 12 hours before bedtime and Sleep outcomes

| Dietary caffeine intake within 12 hours before bedtime | Trouble sleeping  (Yes vs. No)  (OR [95% CI]) | | | Sleep duration  (< 6 hours vs. 6-9 hours)  (OR [95% CI]) | | |
| --- | --- | --- | --- | --- | --- | --- |
|  | Model 1 | Model 2 | Model 3 | Model 1 | Model 2 | Model 3 |
| No pre-bed intake | 1.20^*^  (1.05,1.37) | 0.96  (0.82,1.13) | 0.96  (0.82,1.12) | 0.85^*^  (0.73,1.00) | 1.06  (0.89,1.26) | 1.06  (0.89,1.27) |
| 1-50 mg/day | 1.22^*^  (1.10,1.36) | 1.03  (0.90,1.17) | 1.02  (0.90,1.17) | 0.78^*^  (0.68,0.89) | 1.00  (0.86,1.16) | 1.01  (0.86,1.17) |
| 51-100 mg/day | 1.21^*^  (1.06,1.38) | 1.04  (0.89,1.20) | 1.02  (0.88,1.19) | 0.91  (0.77,1.08) | 1.13  (0.93,1.37) | 1.13  (0.93,1.37) |
| 101-200 mg/day | 1.19^*^  (1.05,1.35) | 0.95  (0.82,1.10) | 0.95  (0.82,1.10) | 0.92  (0.80,1.07) | 1.16  (0.99,1.35) | 1.16  (0.99,1.36) |
| 201-400 mg/day | 1.47^*^  (1.24,1.73) | 1.11  (0.91,1.35) | 1.11  (0.90,1.35) | 0.97  (0.77,1.21) | 1.13  (0.89,1.45) | 1.14  (0.89,1.45) |
| > 400 mg/day | 1.85^*^  (1.36,2.51) | 1.13  (0.83,1.53) | 1.16  (0.85,1.56) | 1.36  (0.86,2.16) | 1.36  (0.79,2.35) | 1.40  (0.81,2.42) |

Note: OR = odds ratio; CI = confidence interval. Dietary caffeine intake within 12 hours before bedtime is the exposure variable, and the reference group is no daily caffeine intake (0 mg/day). Sleep outcomes were modeled separately. The reference level for sleep duration is (6-9 hours), the reference level for trouble sleeping is ‘No’. Model 1 was unadjusted and included dietary caffeine intake as the independent variable only. Model 2 was adjusted for age (continuous), sex, race, education, PIR, smoking status, alcohol consumption, depressive symptoms, and diabetes. Model 3 was additionally adjusted for heart failure.

* indicate statistical significance (95% CI excluding 1).

**Table S3.** Associations between Caffeine Intake within 8 hours before bedtime and Sleep outcomes

| Dietary caffeine intake within 8 hours before bedtime | Trouble sleeping  (Yes vs. No)  (OR [95% CI]) | | | Sleep duration  (< 6 hours vs. 6-9 hours)  (OR [95% CI]) | | |
| --- | --- | --- | --- | --- | --- | --- |
|  | Model 1 | Model 2 | Model 3 | Model 1 | Model 2 | Model 3 |
| No pre-bed intake | 1.23^*^  (1.11,1.38) | 1.01  (0.89,1.16) | 1.01  (0.88,1.16) | 0.79^*^  (0.69,0.91) | 0.99  (0.85,1.15) | 0.99  (0.85,1.15) |
| 1-50 mg/day | 1.25^*^  (1.12,1.38) | 1.04  (0.91,1.19) | 1.03  (0.90,1.19) | 0.80^*^  (0.69,0.92) | 1.03  (0.89,1.20) | 1.04  (0.90,1.21) |
| 51-100 mg/day | 1.19^*^  (1.03,1.38) | 0.98  (0.84,1.15) | 0.97  (0.83,1.13) | 0.98  (0.82,1.17) | 1.17  (0.96,1.42) | 1.18  (0.97,1.43) |
| 101-200 mg/day | 1.21^*^  (1.03,1.41) | 0.94  (0.80,1.10) | 0.94  (0.80,1.11) | 1.10  (0.91,1.33) | 1.35^*^  (1.08,1.68) | 1.36^*^  (1.09,1.69) |
| 201-400 mg/day | 1.39^*^  (1.12,1.72) | 1.01  (0.77,1.32) | 1.01  (0.77,1.32) | 1.42^*^  (1.07,1.90) | 1.59^*^  (1.16,2.18) | 1.60^*^  (1.16,2.19) |
| > 400 mg/day | 1.02  (0.59,1.75) | 0.52  (0.27,1.02) | 0.53  (0.27,1.03) | 1.04  (0.50,2.13) | 0.99  (0.45,2.17) | 0.98  (0.44,2.17) |

Note: OR = odds ratio; CI = confidence interval. Dietary caffeine intake within 8 hours before bedtime is the exposure variable, and the reference group is no daily caffeine intake (0 mg/day). Sleep outcomes were modeled separately. The reference level for sleep duration is (6-9 hours), the reference level for trouble sleeping is ‘No’. Model 1 was unadjusted and included dietary caffeine intake as the independent variable only. Model 2 was adjusted for age (continuous), sex, race, education, PIR, smoking status, alcohol consumption, depressive symptoms, and diabetes. Model 3 was additionally adjusted for heart failure.

* indicate statistical significance (95% CI excluding 1).

**Table S4.** Age interaction models for associations between Caffeine Intake within 12 hours before bedtime and Sleep outcomes

| Exposures | Trouble sleeping  (Yes vs. No)  (OR [95% CI]) | | | Sleep duration  (< 6 hours vs. 6-9 hours)  (OR [95% CI]) | | |
| --- | --- | --- | --- | --- | --- | --- |
|  | Model 1 | Model 2 | Model 3 | Model 1 | Model 2 | Model 3 |
| No pre-bed intake  x Aged 40-64 | 0.92 (0.66,1.28) | 1.02 (0.72,1.44) | 1.04 (0.73,1.47) | 0.93 (0.67,1.28) | 1.19 (0.81,1.74) | 1.19 (0.82,1.72) |
| 1-50 mg/day  x Aged 40-64 | 1.04 (0.81,1.34) | 1.10 (0.83,1.45) | 1.12 (0.84,1.49) | 0.93 (0.67,1.29) | 1.21 (0.83,1.75) | 1.20 (0.82,1.74) |
| 51-100 mg/day  x Aged 40-64 | 0.87 (0.66,1.15) | 0.96 (0.71,1.32) | 1.01 (0.74,1.37) | 0.82 (0.57,1.18) | 1.02 (0.68,1.53) | 1.02 (0.68,1.54) |
| 101-200 mg/day  x Aged 40-64 | 0.80 (0.60,1.07) | 0.96 (0.70,1.33) | 1.00 (0.72,1.38) | 0.89 (0.63,1.27) | 1.35 (0.92,1.97) | 1.34 (0.91,1.97) |
| 201-400 mg/day  x Aged 40-64 | 0.95 (0.66,1.38) | 1.18 (0.77,1.82) | 1.20 (0.78,1.86) | 1.04 (0.67,1.63) | 1.33 (0.81,2.17) | 1.34 (0.82,2.19) |
| > 400 mg/day  x Aged 40-64 | 0.89 (0.43,1.85) | 0.87 (0.36,2.12) | 0.79 (0.34,1.86) | 1.15 (0.49,2.72) | 1.01 (0.39,2.63) | 0.91 (0.36,2.29) |
| No pre-bed intake  x Aged ≥ 65 | 1.04 (0.73,1.49) | 1.18 (0.82,1.71) | 1.22 (0.84,1.76) | 1.25 (0.86,1.82) | 1.41 (0.94,2.11) | 1.42 (0.95,2.14) |
| 1-50 mg/day  x Aged ≥ 65 | 1.10 (0.82,1.47) | 1.08 (0.76,1.52) | 1.11 (0.78,1.58) | 1.07 (0.76,1.52) | 1.13 (0.76,1.68) | 1.13 (0.76,1.69) |
| 51-100 mg/day  x Aged ≥ 65 | 0.92 (0.63,1.34) | 1.02 (0.67,1.54) | 1.06 (0.70,1.61) | 0.89 (0.61,1.30) | 0.97 (0.62,1.50) | 0.99 (0.64,1.55) |
| 101-200 mg/day  x Aged ≥ 65 | 0.78 (0.52,1.16) | 0.93 (0.60,1.44) | 0.99 (0.64,1.54) | 0.96 (0.61,1.52) | 1.28 (0.79,2.09) | 1.30 (0.79,2.14) |
| 201-400 mg/day  x Aged ≥ 65 | 0.83 (0.50,1.38) | 1.01 (0.56,1.81) | 1.03 (0.57,1.87) | 1.03 (0.51,2.09) | 1.02 (0.43,2.41) | 1.04 (0.45,2.45) |
| > 400 mg/day  x Aged ≥ 65 | 1.46 (0.55,3.89) | 1.34 (0.41,4.37) | 1.22 (0.39,3.78) | 1.00 (0.28,3.54) | 0.58 (0.13,2.54) | 0.53 (0.13,2.28) |

Note: OR = odds ratio; CI = confidence interval. Dietary caffeine intake within 12 hours before bedtime is the exposure variable, and the reference group is no daily caffeine intake (0 mg/day). The reference level of the age group is ‘aged 18-39’. Sleep outcomes were modeled separately. The reference level for sleep duration is (6-9 hours), the reference level for trouble sleeping is ‘No’. Model 1 was unadjusted and included dietary caffeine intake as the independent variable only. Model 2 was adjusted for sex, race, education, PIR, smoking status, alcohol consumption, depressive symptoms, and diabetes. Model 3 was additionally adjusted for heart failure.

95% CI excluding 1 indicates statistical significance.

**Table S5.** Age interaction models for associations between Caffeine Intake within 8 hours before bedtime and Sleep outcomes

| Exposures | Trouble sleeping  (Yes vs. No)  (OR [95% CI]) | | | Sleep duration  (< 6 hours vs. 6-9 hours)  (OR [95% CI]) | | |
| --- | --- | --- | --- | --- | --- | --- |
|  | Model 1 | Model 2 | Model 3 | Model 1 | Model 2 | Model 3 |
| No pre-bed intake  x Aged 40-64 | 0.94  (0.70,1.26) | 1.09  (0.80,1.47) | 1.09  (0.80,1.49) | 0.97  (0.70,1.34) | 1.30  (0.90,1.89) | 1.31  (0.91,1.89) |
| 1-50 mg/day  x Aged 40-64 | 1.08  (0.83,1.40) | 1.18  (0.88,1.58) | 1.21  (0.90,1.63) | 0.88  (0.64,1.19) | 1.15  (0.82,1.62) | 1.13  (0.81,1.60) |
| 51-100 mg/day  x Aged 40-64 | 0.85  (0.63,1.14) | 0.97  (0.71,1.33) | 0.99  (0.72,1.38) | 0.81  (0.56,1.16) | 0.98  (0.62,1.55) | 0.97  (0.61,1.53) |
| 101-200 mg/day  x Aged 40-64 | 0.85  (0.58,1.23) | 1.09  (0.70,1.68) | 1.10  (0.71,1.71) | 0.94  (0.63,1.41) | 1.22  (0.77,1.94) | 1.23  (0.77,1.95) |
| >200 mg/day  x Aged 40-64 | 1.02  (0.56,1.84) | 1.18  (0.60,2.34) | 1.20  (0.60,2.41) | 1.12  (0.65,1.90) | 1.27  (0.71,2.28) | 1.28  (0.71,2.30) |
| No pre-bed intake  x Aged ≥ 65 | 1.02  (0.74,1.41) | 1.17  (0.83,1.64) | 1.18  (0.84,1.66) | 1.17  (0.83,1.66) | 1.31  (0.91,1.90) | 1.34  (0.92,1.94) |
| 1-50 mg/day  x Aged ≥ 65 | 1.03  (0.74,1.44) | 1.07  (0.72,1.59) | 1.11  (0.74,1.66) | 1.06  (0.74,1.50) | 1.16  (0.77,1.75) | 1.16  (0.76,1.75) |
| 51-100 mg/day  x Aged ≥ 65 | 0.73  (0.49,1.08) | 0.82  (0.53,1.28) | 0.85  (0.55,1.32) | 0.90  (0.60,1.35) | 1.00  (0.61,1.64) | 1.01  (0.61,1.66) |
| 101-200 mg/day  x Aged ≥ 65 | 0.68  (0.43,1.08) | 0.97  (0.58,1.62) | 1.00  (0.60,1.67) | 0.89  (0.52,1.52) | 1.01  (0.55,1.86) | 1.03  (0.56,1.90) |
| > 200 mg/day  x Aged ≥ 65 | 1.79  (0.83,3.90) | 2.13  (0.82,5.51) | 2.15  (0.80,5.77) | 1.30  (0.44,3.83) | 1.20  (0.32,4.46) | 1.21  (0.32,4.50) |

Note: OR = odds ratio; CI = confidence interval. Dietary caffeine intake within 8 hours before bedtime is the exposure variable, and the reference group is no daily caffeine intake (0 mg/day). The 201–400 and >400 mg/day categories were combined because of insufficient sample size in the >400 mg/day group across age groups. The reference level of the age group is ‘aged 18-39’. Sleep outcomes were modeled separately. The reference level for sleep duration is (6-9 hours), the reference level for trouble sleeping is ‘No’. Model 1 was unadjusted and included dietary caffeine intake as the independent variable only. Model 2 was adjusted for sex, race, education, PIR, smoking status, alcohol consumption, depressive symptoms, and diabetes. Model 3 was additionally adjusted for heart failure. 95% CI excluding 1 indicates statistical significance.

**Table S6.** Associations between Total Dietary Caffeine Intake and Sleep outcomes

| Total dietary caffeine intake | Trouble sleeping  (Yes vs. No)  (OR [95% CI]) | | | Sleep duration  (< 6 hours vs. 6-9 hours)  (OR [95% CI]) | | |
| --- | --- | --- | --- | --- | --- | --- |
|  | Model 1 | Model 2 | Model 3 | Model 1 | Model 2 | Model 3 |
| Low  (1-99mg/day) | 1.11 (0.95,1.30) | 1.03  (0.84,1.27) | 1.00  (0.81,1.25) | 0.92  (0.76,1.12) | 1.19  (0.98,1.45) | 1.17  (0.97,1.43) |
| Moderate  (100-399 mg/day) | 1.33^*^(1.15,1.53) | 1.06  (0.90,1.25) | 1.04  (0.88,1.23) | 0.81  (0.66,1.00) | 1.12  (0.89,1.40) | 1.10  (0.88,1.39) |
| High  (≥ 400mg/day) | 1.59^*^(1.35,1.88) | 1.11  (0.89,1.38) | 1.06  (0.84,1.33) | 1.21  (0.91,1.60) | 1.56^*^  (1.12,2.17) | 1.46^*^  (1.04,2.06) |

Note: OR = odds ratio; CI = confidence interval. Dietary caffeine intake is the exposure variable, the reference level is ‘No Caffeine (0 mg/day)’. Sleep outcomes were modeled separately (N = 20,986 for trouble sleeping, N = 19,317 for sleep duration). The reference level for sleep duration is (6-9 hours), the reference level for trouble sleeping is ‘No’. Model 1 was unadjusted and included dietary caffeine intake as the independent variable only. Model 2 was adjusted for age (continuous), sex, race, education, PIR, smoking status, alcohol consumption, depressive symptoms, diabetes, and heart failure. Model 3 was additionally adjusted for daytime sleepiness.

* indicate statistical significance (95% CI excluding 1).

**Table S7.** Associations between Caffeine Intake within 12 hours before bedtime and Sleep outcomes

| Dietary caffeine intake within 12 hours before bedtime | Trouble sleeping  (Yes vs. No)  (OR [95% CI]) | | | Sleep duration  (< 6 hours vs. 6-9 hours)  (OR [95% CI]) | | |
| --- | --- | --- | --- | --- | --- | --- |
|  | Model 1 | Model 2 | Model 3 | Model 1 | Model 2 | Model 3 |
| No pre-bed intake | 1.24^*^  (1.02,1.52) | 0.99  (0.79,1.26) | 0.99  (0.78,1.26) | 0.88  (0.68,1.13) | 1.14  (0.86,1.51) | 1.14  (0.86,1.50) |
| 1-50 mg/day | 1.25^*^  (1.08,1.45) | 1.08  (0.88,1.31) | 1.05  (0.86,1.28) | 0.84  (0.68,1.04) | 1.16  (0.93,1.45) | 1.14  (0.91,1.42) |
| 51-100 mg/day | 1.28^*^  (1.08,1.51) | 1.08  (0.88,1.34) | 1.06  (0.85,1.31) | 0.98  (0.76,1.27) | 1.27  (0.96,1.68) | 1.26  (0.96,1.67) |
| 101-200 mg/day | 1.22^*^  (1.03,1.44) | 0.96  (0.78,1.18) | 0.92  (0.73,1.15) | 0.95  (0.75,1.19) | 1.23  (0.97,1.57) | 1.20  (0.93,1.54) |
| 201-400 mg/day | 1.55^*^  (1.25,1.93) | 1.24  (0.94,1.63) | 1.21  (0.91,1.60) | 0.92  (0.67,1.27) | 1.12  (0.79,1.60) | 1.08  (0.75,1.55) |
| > 400 mg/day | 1.73^*^  (1.15,2.61) | 1.25  (0.82,1.88) | 1.12  (0.74,1.72) | 1.05  (0.55,2.02) | 1.23  (0.55,2.77) | 1.13  (0.49,2.58) |

Note: OR = odds ratio; CI = confidence interval. Dietary caffeine intake within 12 hours before bedtime is the exposure variable, and the reference group is no daily caffeine intake (0 mg/day). Sleep outcomes were modeled separately. The reference level for sleep duration is (6-9 hours), the reference level for trouble sleeping is ‘No’. Model 1 was unadjusted and included dietary caffeine intake as the independent variable only. Model 2 was adjusted for age (continuous), sex, race, education, PIR, smoking status, alcohol consumption, depressive symptoms, diabetes, and heart failure. Model 3 was additionally adjusted for daytime sleepiness.

* indicate statistical significance (95% CI excluding 1).

**Table S8.** Associations between Caffeine Intake within 8 hours before bedtime and Sleep outcomes

| Dietary caffeine intake within 8 hours before bedtime | Trouble sleeping  (Yes vs. No)  (OR [95% CI]) | | | Sleep duration  (< 6 hours vs. 6-9 hours)  (OR [95% CI]) | | |
| --- | --- | --- | --- | --- | --- | --- |
|  | Model 1 | Model 2 | Model 3 | Model 1 | Model 2 | Model 3 |
| No pre-bed intake | 1.34^*^  (1.14,1.58) | 1.11  (0.91,1.36) | 1.09  (0.89,1.34) | 0.85  (0.68,1.07) | 1.11  (0.88,1.40) | 1.09  (0.87,1.37) |
| 1-50 mg/day | 1.24^*^  (1.07,1.43) | 1.03  (0.85,1.26) | 1.01  (0.82,1.23) | 0.82  (0.66,1.02) | 1.15  (0.91,1.45) | 1.13  (0.90,1.43) |
| 51-100 mg/day | 1.20  (1.00,1.44) | 1.00  (0.81,1.24) | 0.96  (0.76,1.21) | 0.98  (0.73,1.31) | 1.19  (0.88,1.61) | 1.18  (0.87,1.61) |
| 101-200 mg/day | 1.24^*^  (1.02,1.51) | 0.97  (0.79,1.21) | 0.94  (0.75,1.17) | 1.22  (0.93,1.61) | 1.63^*^  (1.19,2.23) | 1.58^*^  (1.15,2.17) |
| 201-400 mg/day | 1.53^*^  (1.15,2.03) | 1.22  (0.85,1.76) | 1.17  (0.83,1.65) | 1.30  (0.86,1.95) | 1.50  (0.91,2.47) | 1.46  (0.88,2.42) |
| > 400 mg/day | 0.96  (0.50,1.87) | 0.62  (0.29,1.33) | 0.52  (0.24,1.13) | 0.85  (0.36,2.03) | 1.07  (0.39,2.98) | 0.94  (0.31,2.83) |

Note: OR = odds ratio; CI = confidence interval. Dietary caffeine intake within 8 hours before bedtime is the exposure variable, and the reference group is no daily caffeine intake (0 mg/day). Sleep outcomes were modeled separately. The reference level for sleep duration is (6-9 hours), the reference level for trouble sleeping is ‘No’. Model 1 was unadjusted and included dietary caffeine intake as the independent variable only. Model 2 was adjusted for age (continuous), sex, race, education, PIR, smoking status, alcohol consumption, depressive symptoms, diabetes, and heart failure. Model 3 was additionally adjusted for daytime sleepiness.

* indicate statistical significance (95% CI excluding 1).
